# Determinants of early discharge after birth among mothers delivered vaginally at health facilities: further analysis of the Ethiopian demographic health survey

**DOI:** 10.1186/s12889-023-16922-y

**Published:** 2023-10-30

**Authors:** Desalegn Anmut Bitew, Mengistie Diress, Yibeltal Yismaw Gela, Daniel Gashaneh Belay, Anteneh Ayelign Kibret, Dagmawi Chilot, Deresse Sinamaw, Mohammed Abdu Seid, Abdulwase Mohammed Seid, Wudneh Simegn, Habitu Birhan Eshetu, Amare Agmas Andualem

**Affiliations:** 1 Department of Reproductive Health, Institute of Public Health, College of Medicine and Health Sciences, University of Gondar, P. O. Box 196, Gondar, Ethiopia; 2 Department of Human Physiology, School of Medicine, College of Medicine and Health Sciences, University of Gondar, P. O. Box 196, Gondar, Ethiopia; 3 Department of Human Anatomy, School of Medicine, College of Medicine and Health Sciences, University of Gondar, P. O. Box 196, Gondar, Ethiopia; 4 Department of Epidemiology and Biostatics, Institute of Public Health, College of Medicine and Health Sciences, University of Gondar, P. O. Box 196, Gondar, Ethiopia; 5 College of Health Sciences, Center for Innovative Drug Development and Therapeutic Trials for Africa (CDT-Africa), Addis Ababa University, Addis Ababa, Ethiopia; 6 Department of Biomedical Science, Debre Markos University, P. O. Box 269, Debre Markos, Ethiopia; 7 Unit of Human Physiology, Department of Biomedical Science, College of Health Sciences, Debre Tabor University, P. O. Box: 272, Debre Tabor, Ethiopia; 8 Department of Clinical Pharmacy, University of Gondar, P. O. Box 196, Gondar, Ethiopia; 9 Department of Social and Administrative Pharmacy, University of Gondar, P. O. Box 196, Gondar, Ethiopia; 10 Department of Health Education and Behavioral Sciences, University of Gondar, P. O. Box 196, Gondar, Ethiopia; 11 Department of Anesthesia, Wollo University, P. O. Box, 1145, Dessie, Ethiopia

**Keywords:** Early discharge, Determinants, Birth, Ethiopia

## Abstract

**Introduction:**

The majority of maternal and newborn deaths take place during the first few hours and days after birth and thus postnatal contacts should begin as early as possible, especially within the first 24 h, then again within two to three days after delivery. Globally, early postnatal discharge has increased over the past 50 years and currently too. Even if Ethiopia has very low PNC coverage, there is no evidence on who is discharged early. Hence, the aim of this study was to determine the magnitude and the predictors for early postnatal discharge in Ethiopia.

**Methods:**

This study was based on the secondary data analysis using the Ethiopian Demographic and Health survey (EDHS) 2016 data set. The weighted sample of 2,225 delivered mothers were included for the final analysis. The model was best fitted as assessed by Hosmer-Lemeshow test (p value = 0.1988). The variables with P-value ≤ 0.2 in the bi- variable binary logistic regression analysis were included in to the multi-variable binary logistic regression analysis. The Adjusted Odds Ratio (AOR) with 95% confidence interval (95% CI) was computed to assess the strength of association between the outcome and independent variables. The variables with a *P*-value of less than 0.05 in the multi-variable binary logistic regression analysis were declared as statistically significant predictors of the outcome variable.

**Result:**

The overall magnitude of early discharge was 70.41% (CI: 68.48, 72.30). Residence (rural; AOR: 0.61, 95% CI: 0.46, 0.80), educational status (No education; AOR: 1.87, 95% CI: 1.19, 2.94), religion (Muslim; AOR: 0.69, 95% CI: 0.55, 0.87, Others; AOR: 0.24, 95% CI: 0.10, 0.57), wealth index (Poor; AOR: 0.77; 95% CI: 0.59, 0.99), marital status (Not married; AOR: 0.29; 95% CI: 0.13, 0.67), ANC visits (No ANC visits; AOR: 0.63; 95% CI: 0.46,0.86), parity (3rd parity; AOR: 1.48; 95% CI: 1.03, 2.11), and size of the child (larger size; AOR: 0.63;95% CI: 0.50,0.79, (smaller size; AOR: 0.72; 95% CI: 0.56,0.92) were independent determinants of early discharge.

**Conclusion:**

A substantial proportions of mothers in Ethiopia had been discharged early (before 24 h). Residence, education, wealth index, religion, marital status, ANC follow up, parity and size of the child were predictors of early discharge. Adequate hospital stay should be promoted. Since the early discharge in Ethiopia is very high, home based postnatal visit should be strengthened focusing the identified predictors.

## Introduction

Duration of postnatal hospital stay has declined rapidly in the past three/ four decades [1]. Globally, there is variation in postnatal length of stay (LoS). In spite of an increase in medical interventions during pregnancy and childbirth, there is a reduction in the LoS in the facility after child birth for women and infants. Many high income counties, such as the United Kingdom, Australia, and Canada, have an average stay of 1.5, 2.8, and 1.7 days, respectively [2]. There is some evidence suggesting that low-risk women and babies are being discharged from 4 to 6 h following birth [3, 4].

Early hospital discharge after child birth generally refers to the postpartum hospital discharge of the mother and newborn within 48 hours [5]. The cut points on the duration on early discharge across countries varies from 12 to 72 hours [6]. Newborns’ and Mothers’ Health Protection Act (NMHPA) of United States government’ in 1996 ensures coverage of a hospital stay for 48 h following vaginal birth for parents [7]. The Spanish Association of Pediatrics also recommended a discharge from the hospital after 48 h following birth for healthy newborns [8]. The World Health Organization (WHO) recommends healthy mothers and newborns to stay and receive care at the facility for at least 24 h following an uncomplicated vaginal delivery at a health facility [9]. Because the majority of maternal and newborn deaths take place during the first few hours and days after birth, postnatal contacts should begin as early as possible in the postnatal period, especially within the first 24 h, then again within two to three days after delivery [10].

The large gap in postnatal care coverage is evident in a recent analysis of Demographic and Health Surveys in 23 African countries. Approximately, one-third of women in sub-Saharan Africa give birth in facilities, and less than 13% receive a postnatal care visit within two days of delivery [10, 11]. Early postnatal care is needed to encourage preventive behaviors and practices, to increase the likelihood that potentially life-threatening complications are detected, referred, and treated as early as possible and to provide the mother with important information on caring for herself and her baby [12].

The adverse outcomes of early discharge includes insufficient time for women and babies to establish breastfeeding [13, 14], delay in the identification and treatment of maternal and infant complications (jaundice, dehydration, infections), increased infant and maternal readmissions within 28 days, decreased maternal confidence due to lack of professional support, less maternal satisfaction with postnatal hospital care, and increased prevalence of maternal depression [14–18]. On the contrary, others suggested that early discharge from health facility creates opportunities for family-centered care, creates greater opportunities for families to bond in their home environment and is a safe and cost-effective way to provide postnatal care [19, 20].

The 2016 EDHS found that among women age 15–49 giving birth, 17% had a postnatal check during the first 2 days after birth. Four in five women (81%) did not receive a postnatal check [21]. Even though global early postnatal discharge has increased over the past 50 years and today, and Ethiopia has very low PNC coverage, there is no evidence on “what proportions of mothers are being discharged early and what are the predictors of early postnatal discharge?” The main objective of this study was determining the magnitude of early postnatal discharge and to identify potential predictors for early postnatal discharge in Ethiopia. The evidence from this study will allow policy makers, program mangers and clinicians to improve quality of postnatal care. It will also give an insight for researchers to investigate some controversial findings.

## Methods

### Study setting, study design, period

This was a cross-sectional study conducted in Ethiopia based on the fourth Ethiopian demographic and health survey data (EDHS, 2016). The EDHS 2016 was conducted from January 18 to June 27, 2016 [21]. Ethiopia is the second most populous country in Africa which is federally decentralized into ten regions and two city administrations [21] and ithas a total estimated **118,977,453** population [22]. The 2016 EDHS was cross-sectional by design. Secondary data analysis was performed using evidence from the EDHS 2016 data set which is the latest national survey conducted in nine regional states and two administrative cities. The EDHS 2016 was based on 645 enumeration areas.

### Data source, study population and sampling

The sample for DHS survey was designed to represent all regions and administrative cities in the country. The survey participants were selected using stratified and two stage sampling methods: enumeration areas (EAs) in the first stage and households in the second stage. Each region was stratified into urban and rural areas. Then probability proportional allocation to sample size was made. For the 2016 DHS, 645 enumeration areas (EAs) were selected. From this, 202 EAs were from urban and 443 were from rural areas. We have used individual record (IR) data set of EDHS 2016 for this study. The data was accessed from measure DHS website (http://www.measuredhs.com) A total of 2,225 weighted mothers who gave birth vaginally were included for the final analysis. All the frequencies and the percentages in the result section were weighted.

**Fig. 1:**
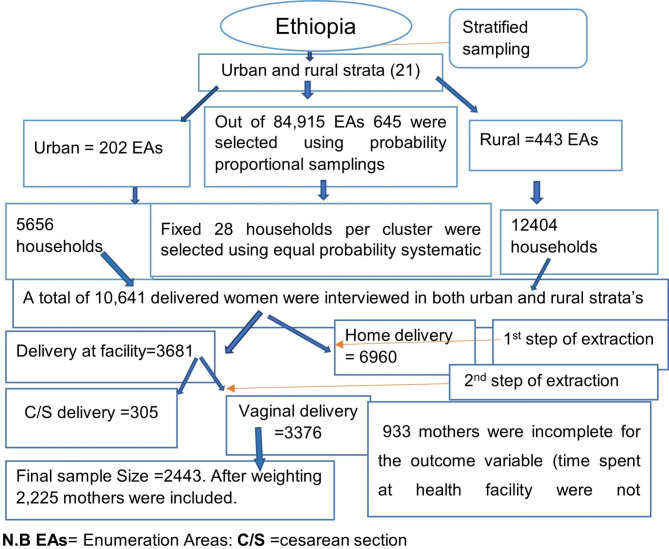
Schematic representation of the sampling procedures in the study of early discharge after child birth and associated factors (N = 2,225: weighted), Ethiopia, 2016

The summary of the sampling technique was described as follows (Fig. 1).

### Variables and measurements

The outcome variable was early discharge after vaginal birth. It was dichotomized as (yes/no). In EDHS, women who gave birth in health facilities were asked ***‘How long after (NAME of the new born) was delivered did you stay there?’*** The responses were recorded in hours (if less than a day), days (if less than a week) and weeks (if less than a month). We dichotomized the outcome variable based on the WHO recommendation as early discharge (yes) if length of stay in the health facility was < 24 h and not early discharge (No) if length of stay was ≥ 24 h) [23].

The independent variables were socio-demographic characteristics including the maternal age, educational level, marital status, place of residence, wealth index, religion, and working status and obstetric characteristics like place of delivery, pregnancy type, size of the child, parity, gestational age, wantedness of pregnancy, history of abortion, antenatal care utilization and gestational age.

#### Wealth index

In the dataset, the categories for wealth index were presented as Poorest, Poorer, Middle, Richer, and Richest [21]. In our study, a new variable was generated which had three categories “Poor”, “Middle” and “Rich” by merging poorest with poorer and richest with richer.

#### Religion

In the 2016 EDHS, religion had subcategories of Orthodox, Muslim, Protestant, Catholic, traditional followers and others [21]. In our study, the former three were encoded independently and Catholic and traditional religion followers were merged to others category.

### Data processing and analysis

This study used the extracted data from EDHS 2016 individual record (IR file) folder. The data extraction and analysis was done using STATA version 14 software. Before analysis, data was cleaned using frequency; listing and sorting to identify any missed values. The time spent at health facility was incomplete for 933 mothers and were excluded. The model fitness was assessed using Hosmer-Lemeshow test. It was best fitted (p value = 0.1988). Variables with p-value ≤ 0.2 in the bi-variable binary logistic regression analysis were included in to the multi-variable binary logistic regression analysis. The Adjusted Odds Ratio (AOR) with 95% confidence interval (95% CI) was computed to determine the association between early discharge and the independent variables. Variables with a *P*-value of less than 0.05 in the multi variable binary logistic regression analysis were declared as statistically significant predictors of the outcome variable.

## Result

### Socio-demographic characteristics of mothers

Two thousand two hundred twenty five (2,225) mothers were included in this study. About 30% of mothers were within the age range of 25 to 29 years. More than two third(68.50%) of the mothers were rural residents. The educational status of study participants ranges from 7.3% (higher education) to 41.20% (no education). Nearly half (48.10%) of mothers were orthodox followers. Virtually all (99%) of the mothers were married. Almost half (49.12%) of the mothers were not working (Table 1).

**Table 1:** The socio-demographic characteristics of mothers in the study of early discharge after child birth and associated factors (N = 2,225: weighted), Ethiopia, 2022

Variables	Categories	Frequency (%)	Early discharge	
			No (%)	Yes (%)
Age	15–19	130 (5.87)	52 (40.20)	78 (59.80)
	20–24	543 (24.41)	173 (31.80)	370 (68.20)
	25–29	685 (30.76)	168 (24.53)	517 (75.47)
	30–34	439 (19.73)	145 (32.95)	294 (67.05)
	35–39	306 (13.74)	89 (29.0)	217 (71.0)
	40–44	92 (4.10)	19 (20.42)	73 (79.58)
	45–49	30 (1.35)	13 (43.79)	17 (56.21)
Residence	Urban	700 (31.50)	159 (22.68)	541 (77.32)
	Rural	1,525 (68.50)	500 (32.76)	1,025 (67.24)
Education	No education	917 (41.20)	252 (27.50)	665 (72.50)
	Primary	830 (37.30)	282 (34.00)	548 (66.00)
	Secondary	315 (14.14)	77 (24.40)	238 (75.60)
	Higher	163 (7.30)	47 (28.80)	116 (71.20)
Religion	Orthodox	1,070 (48.10)	266 (24.80)	804 (75.20)
	Protestant	430 (19.30)	132 (30.77)	298 (69.23)
	Muslim	700 (31.48)	246 (35.10)	454 (64.90)
	**Others**	25 (1.11)	15 (58.60)	10 (41.40)
Wealth index	Poor	575 (25.84)	204 (35.56)	371(64.44)
	Middle	398 (17.88)	123 (30.87)	275 (69.13)
	Rich	1,252 (56.27)	331 (26.44)	921 (73.56)
Marital status	Not married	27 (1.20)	16 (59.05)	11 (40.95)
	Married	2,198 (98.78)	642 (29.20)	1,556 (70.80)
Working status	Not working	1,093 (49.12)	355 (32.45)	738 (67.55)
	Working	1,132 (50.88)	304 (26.83)	828 (73.17)

Other*= catholic, traditional and other local beliefs; N: Total number of study participants

### Obstetrics characteristics of mothers

More than half(55.28%) of the mothers had at least 4 antenatal care (ANC) visits. About 68% of the mothers had parity of less than four. Nearly 96% of mothers gave birth in public health facilities. About 92% of the mothers had history of abortion (Table 2).

**Table 2:** The obstetrics characteristics of mothers in the study of early discharge after child birth and associated factors (N = 2,225: weighted), Ethiopia, 2022

Variables	Categories	Frequency (%)	Early discharge	
			No (%)	Yes (%)
ANC visits	No ANC visit	222 (10.00)	94 (42.57)	128 (57.43)
	< 4 visits	773 (34.72)	219 (28.30)	554 (71.70)
	>= 4 visits	1,230 (55.28)	345 (15.5)	885 (39.78)
Parity	< 4	1,505 (67.64)	436 (28.94)	1,069 (71.06)
	4–7	630 (28.31)	197 (31.24)	433 (68.76)
	>=8	90 (4.05)	26 (28.86)	64 (71.14)
Place of delivery	Public health facilities	2,135 (95.94)	632 (29.61)	1,503 (70.39)
	Private health facilities	90 (4.06)	26 (29.09)	64 (70.91)
Size of child	Larger	743 (33.40)	260 (34.97)	483 (65.03)
	Average	941 (42.28)	226 (23.97)	715 (76.03)
	Smaller	541 (24.32)	173 (31.98)	368 (68.02)
Pregnancy type	Single birth	2,184 (98.15)	642 (29.41)	1,542 (70.59)
	Twin birth	41 (1.85)	16 (38.88)	25 (61.12)
Gestational age (in months)	7	5 (0.25)	3 (51.60)	2 (48.40)
	8	33 (1.46)	17 (52.71)	16 (47.29)
	9	2,175 (97.76)	638 (29.32)	1,537 (70.68)
	10	12 (0.53)	1 (5.59)	11 (94.41)
Pregnancy wanted	Then	1,668 (74.98)	500 (30)	1,168 (70)
	Later	393 (17.65)	113 (28.75)	280 (71.25)
	No more	164 (7.37)	45 (27.41)	119 (72.59)
Abortion history	No	2,036 (91.48)	612 (30.06)	1,424 (69.94)
	Yes	189 (8.52)	46 (24.48)	143 (75.52)

Key: N = Number of study participants; **ANC: Ante natal care**

### Magnitude of early discharge (ED)

In this study, the overall magnitude of early discharge after childbirth was 70.41% (**CI: 68.48, 72.30**). However, this magnitude varied across mothers’ characteristics. For example, it was 77.32% in urban and 67.24% in rural. Similarly, the magnitude of early discharge ranged from 66% for primary education to 75% for secondary education. It also varied by marital status (40.95% among not married and 70.80% among married) (Table 1).

ED was 57.43% among mothers who had no ANC visit while it was 71.95% among mothers who had four and more ANC visits. ED also varied by the size of the child. It ranged from 65.03% for larger size to 76.03% for average size of the child (Table 2).

### Factors associated with early discharge

Among thirteen independent variables entered in to multivariable analysis, eight variables (residence, education, religion, wealth index, marital status, ANC visit, parity, and size of the child) were statistically and significantly associated with early discharge.

Mothers from the rural area had a 39% reduced risk of early discharge compared to their urban counter parts (rural; AOR: 0.61, 95% CI: 0.46, 0.80). None educated mothers had 1.87 times higher risk of early discharge compared to their higher education counter parts (No education; AOR: 1.87, 95% CI: 1.19, 2.94). Muslim follower mothers had a 31% reduced risk of early discharge compared to their orthodox counter parts (Muslim; AOR: 0.69, 95% CI: 0.55, 0.87). Others religion follower mothers had 76% reduced risk of early discharge compared to their orthodox counter parts (Others; AOR: 0.24, 95% CI: 0.10, 0.57). Mothers who had no ANC visits had a 37% reduced risk of early discharge compared to mothers who had 4 or more ANC visits (No ANC visits; AOR: 0.63; 95% CI: 0.46, 0.86). Para 3 mothers were 1.48 times more likely to be discharged early compared to mothers who were more than Para 3 (3rd parity; AOR: 1.48; 95% CI: 1.03, 2.11). Mothers who gave birth for larger size child had a 37% reduced risk of early discharge compared to mothers who gave birth for average size child (larger size; AOR: 0.63; 95% CI: 0.50,0.79). Mothers who gave birth for smaller size child had 28% reduced risk of early discharge compared to mothers who gave birth for average size child (smaller size; AOR: 0.72; 95% CI: 0.56,0.92). Mothers who were not married had 71% reduced risk of early discharge compared to married mothers (Not married; AOR: 0.29; 95% CI: 0.13, 0.67). Mothers who were in the poor economic class had 23% reduced risk of early discharge compared to the mothers in the rich economic class (Poor; AOR: 0.77; 95% CI: 0.59, 0.99) (Table 3).

**Table 3:** Factors associated with early discharge after childbirth among mothers delivered at health facilities in Ethiopia (N = 2,225: weighted), 2022

Variables	Categories	COR (95% CI)	AOR (95%CI)
Age	15–19	Ref	Ref
	20–24	1.43 (0.97, 2.13)	1.11(0.73, 1.70)
	25–29	2.06 (1.39, 3.05)	1.12(0.71, 1.77)
	30–34	1.36 (0.91, 2.04)	0.61(0.37, 1.04)
	35–39	1.64 (1.06, 2.51)	0.81(0.47, 1.42)
	40–44	2.61 (1.41, 4.83)	1.12 (0.54, 2.33)
	45–49	0.86 (0.38, 1.92)	0.45 (0.18, 1.14)
Residence	Urban	Ref	Ref
	Rural	0.60 (0.48, 0.74)	**0.61 (0.46, 0.80)** ^**^
Education	No education	1.06 (0.73, 1.54)	1.87 (1.19, 2.94)*
	Primary	0.78 (0.54, 1.13)	1.17(0.76, 1.78)
	Secondary	1.25 (0.81, 1.91)	1.36 (0.86, 2.14)
	Higher	Ref	Ref
Religion	Orthodox	Ref	Ref
	Protestant	0.74 (0.58, 0.95)	0.89 (0.68, 1.17)
	Muslim	0.61 (0.49, 0.75)	0.69 (0.55, 0.87)^*^
	**Others** ^**a**^	0.23 (0.10, 0.52)	0.24 (0.10, 0.57)^*^
Wealth index	Poor	0.65 (0.52, 0.80)	0.77 (0.59, 0.99)^*^
	Middle	0.80 (0.63, 1.03)	1.02(0.76, 1.37)
	Rich	Ref	Ref
Marital status	Not married	0.28 (0.13, 0.61)	**0.29 (0.13, 0.67)** ^*^
	Married	Ref	Ref
Working status	Not working	Ref	Ref
	Working	3.49 (1.62, 7.52)	1.17 (0.96, 1.43)
ANC visits	No ANC visit	0.53 (0.39, 0.70)	**0.63 (0.46,0.86)** ^*^
	< 4 visits	1.87 (1.37 ,2.55)	1.09 (0.88, 1.35)
	>= 4 visits	Ref	Ref
Parity	1st parity	1.75 (1.27, 2.41)	0.69 (0.48,1.0)
	2nd parity	1.33 (1.03, 1.71)	0.88(0.63, 1.22)
	3rd parity	1.98 (1.43, 2.73)	**1.48 (1.03, 2.11)** ^*^
	More parity	Ref	Ref
Size of child	Average	Ref	Ref
	Larger	0.58 (0.47, 0.72)	**0.63 (0.50, 0.79)** ^**^
	Smaller	0.67 (0.53, 0.84)	**0.72 (0.56, 0.92)** ^*^
Pregnancy type	Single birth	Ref	Ref
	Twin birth	0.65 (0.3478062 1.23)	0.58 (0.29, 1.18)
Abortion history	No	Ref	Ref
	Yes	1.32 (0.94, 1.87)	1.26 (0.87, 1.82)

Key: Bold indicate the significant explanatory variables with their adjusted odds ratio and confidence interval, other ^a^ =traditional and catholic, Ref = reference category, * Significant at p < 0.05 and ** Significant at p < = 0.001

COR: Crude Odds Ratio

AOR: Adjusted Odds Ratio

N: Total number of study participants

## Discussion

The primary objective of this study was to determine the magnitude of early discharge (ED) after vaginal delivery in the health facilities and to identify its associated factors.

In our study, the magnitude of ED was 70.41% (CI: 68.48, 72.30). This finding was higher than the study from Ghana [24] in which the 37.6% mothers were discharged within 24 h following birth, Nepal [25] in which early discharge was 61.7% and Beirut [26] in which 64% were discharged early. The observed variation in the magnitude of ED might be due to the difference in the study population. For instance, in our study mothers who gave birth by cesarean section were excluded while they were included in the Nepal, Ghana, and Beirut studies. This is obvious that mothers who gave birth by cesarean section will stay longer in hospital [27]. Generally the highest percentage of early discharge in Ethiopia may be due to the existing facility limitations such as lack of space to stay [28]. As a result, there might be pressure from a health facility to leave early to accommodate other mothers.

The mothers’ residence, educational status, religion, wealth index, marital status, ANC visit, parity, and size of the child were statistically significant determinant factors for early discharge. In our study, mothers from rural and poor economic status stayed longer in health facilities. This finding was supported by other findings from 30 low-and middle-income countries [2]. The possible explanation might be, women in rural areas and who are poor visits health facilities when there is complication. As a result, complicated case takes longer time to recover. But this finding contradicted with the finding in India [29] which stated that women from rural and in the poor economic class stayed shorter due to fear of cost of the services. This difference could be explained by the fact that maternal health services are free of charge in Ethiopia. This contradiction needs further investigation.

Another finding of our study was, women who gave birth for larger and smaller size neonates stayed longer than mothers who gave birth for neonates of average size. This finding was supported by other studies conducted in Ethiopia [30] and south Africa [31] which stated that the length of hospital stay increased proportionally with decreasing birth weight. This might be explained by low birth weight neonates are at risk many complications like hypothermia, apnea, and respiratory distress syndrome [32]. So, mothers with low birth weight neonates may wait longer till their new borns recovered.

In our study non educated mothers stayed shorter duration compared to mothers with higher education. This study was supported by findings from India [33] and Nepal [25] which stated that women who are less educated and come from poor families stayed shorter duration in health facilities. The possible explanation might be non educated mothers are not well informed about the health risks associated with child birth and they lack awareness about the benefit of staying more in the hospital and can’t anticipate further complications [33].

In our study, mothers currently not married stayed longer. This was supported by a study from 92 country [2]. The possible explanation could be not married women have high power of decision on admission and discharge than married women [34].

### Limitation and strength

Using a national survey increases the representativeness of the results. However, our findings were based on survey data which may be influenced by recall bias. In addition to this, the EDHS data didn’t include facility and provider as well as family related variables which greatly affects the duration of stay.

## Conclusion

Despite the fact that the first 24 h after childbirth provide a golden opportunity to tackle most maternal and neonatal morbidities and mortalities, a very large proportions of mothers in Ethiopia were discharged early (before 24 h). Residence, education, wealth index, religion, marital status, ANC follow up, parity and size of the child at birth were identified as the most significant predictors of early discharge. In addition to institutional delivery promotion, adequate hospital stay should be promoted. Since early discharge in Ethiopia is very high, the postnatal visit in the community should be strengthened focusing the identified predictors. Researchers are expected to further investigate the contradicted finding on rural residency and poor socio economic status. In addition, we would like to recommend researchers to conduct qualitative studies to investigate facility and provider as well as family related variables.

## Data Availability

The dataset supporting the conclusions of this article is available in the measure DHS website (http://www.measuredhs.com) upon request and the extracted data is available with the corresponding author.

## Abbreviations

AOR: adjusted Odds Ratio
COR: Crude Odds ratio
CI: Confidence interval
EDHS: Ethiopia Demographic and Health Survey
ED: early discharge
WHO: World Health Organization
